# A Multi-Species Comparison and Evolutionary Perspectives on Ion Regulation in the Antennal Gland of Brachyurans

**DOI:** 10.3389/fphys.2022.902937

**Published:** 2022-06-02

**Authors:** Kuang-Yu Tseng, Jyuan-Ru Tsai, Hui-Chen Lin

**Affiliations:** ^1^ Department of Life Science, Tunghai University, Taichung, Taiwan; ^2^ Center for Ecology and Environment, Tunghai University, Taichung, Taiwan

**Keywords:** evolutionary physiology, brachyurans, antennal gland, NKA activity, ion regulation

## Abstract

Brachyurans inhabit a variety of habitats and have evolved diverse osmoregulatory patterns. Gills, antennal glands and a lung-like structure are important organs of crabs that maintain their homeostasis in different habitats. Species use different processes to regulate ions in the antennal gland, especially those with high terrestriality such as Grapsoidea and Ocypodoidea. Our phylogenetic generalized least square (PGLS) result also suggested that there is a correlation between antennal gland NKA activity and urine-hemolymph ratio for Na^+^ concentration in hypo-osmotic environments among crabs. Species with higher antennal gland NKA activity showed a lower urine-hemolymph ratio for Na^+^ concentration under hypo-osmotic stress. These phenomenon may correlate to the structural and functional differences in gills and lung-like structure among crabs. However, a limited number of studies have focused on the structural and functional differences in the antennal gland among brachyurans. Integrative and systemic methods like next generation sequencing and proteomics method can be useful for investigating the differences in multi-gene expression and sequences among species. These perspectives can be combined to further elucidate the phylogenetic history of crab antennal glands.

## Introduction

Evolutionary physiology uses phylogenetics to describe physiological patterns (Felsenstein, 1985; Garland et al., 1992; Garland and Carter, 1994). When we compare the physiological patterns among species, it is important to acknowledge that species are not independent to one another, and that an underlying phylogenetic relationship connects them all (Felsenstein, 1985). Directly comparing the differences among species is inadequate because being independent and identically distributed (IID) is a prior assumption for parametric statistical analysis (Garland et al., 1993; Gotelli and Ellison, 2004). Previous studies used 1) the topology of phylogenetic trees to adjust the variance between variables and 2) degrees of freedom to remove the non-independent effect and more accurately compare features among species (Felsenstein, 1985; Garland et al., 1992; Garland et al., 1993). For example, Garland et al. (1993) used the Monte Carlo method and gave tree topologies to estimate the 95th percentile of *F* value distribution for the null hypotheses of ANOVA and ANCOVA, then used this *F* value as a threshold for hypothesis testing. This is known as the phylogenetic ANOVA and ANCOVA. In addition, studies used methods such as Moran’s I autocorrelation, which originated from spatial analysis, to justify whether the similarity of traits corresponds with the phylogenetic distance among species (Gittleman and Kot, 1990; Faria et al., 2017). Evolutionary physiology was further extended to quantitative genetics and genome studies to recognize the differences in gene sequences and expressions among species based on phylogenetics (Storz et al., 2015). In their pioneer study, McNamara and Faria (2012) used Pearson’s correlation analysis with phylogenetically independent contrasts (PIC) correction to analyze the relationship between habitat and hemolymph osmolality among palaemonid shrimp. Result showed that type I error can be reduced when the phylogenetic structure has been taken into consideration (McNamara and Faria, 2012). Their result indicated that performing a physiological comparison from a phylogenetic perspective can reduce statistical errors when performing multiple species investigations and connecting the physiological features of species to their phylogenetic history.

Brachyura is a very diverse group found across the world. It is made up of at least 7,250 species in a wide range of habitats, including marine, intertidal, terrestrial and freshwater zones (Péqueux, 1995; Ng et al., 2008; Davie et al., 2015). Several ion regulatory patterns have been reported in this clade (Henry et al., 2012; McNamara and Faria, 2012; Allen and Weihrauch, 2021). Two important ion regulatory organs—gills and antennal glands—have different functional patterns in different species. Additionally, some species have well-developed lung-like structure which can facilitate the oxygen exchange in terrestrial areas (Greenaway and Farrelly, 1984; Innes and Taylor, 1986; Tsai and Lin, 2012). Along with organ morphology and function, two previous studies outlined the phylogenetic relationships among brachyuran species (Tsang et al., 2014; Ma et al., 2019). This gives us the opportunity to analyze and describe the ion regulatory patterns among crabs from a phylogenetic perspective. Previous reviews and studies focused efforts on identifying the function and structure of gills in crabs (Kirschner, 2004; Weihrauch et al., 2004; Freire et al., 2008; Charmantier et al., 2009; Henry et al., 2012; McNamara and Faria, 2012; Weihrauch et al., 2017; Weihrauch and Allen, 2018; Allen and Weihrauch, 2021) and several important reviews have laid out more details on the structure and ion regulation mechanism of antennal gland of brachyurans (Weihrauch et al., 2004; Freire et al., 2008; Charmantier et al., 2009). In this review, we follow these studies and focus on comparing the structure and ion regulation in the antennal gland among crab species in different clades and habitats. First, we will briefly introduce the habitats and phylogeny of brachyurans, then discuss the differences in the ion regulatory patterns of species’ antennal glands and give an integrated perspectives and introduce questions about the ion regulatory patterns in the antennal gland among brachyurans.

## Habitats and Phylogenetic Relationships Among Brachyuran Species

The phylogenetic relationships and habitat diversity of brachyuran crabs interweave to form a wide spectrum of osmoregulatory and ion regulatory patterns among different species. Here we introduce what is currently understood of the habitat properties and phylogenetic relationships among brachyurans. Tsang et al. (2014) included 142 crab species in 58 families in an investigation on the phylogeny of brachyurans using six chromosomal genes and two mitochondrial genes. Four sections belonged to brachyuran—Dromiacea, Raninoida, Cyclodorippoida and Eubrachyura—and there were two monophyletic subsections in Eubrachyura—Heterotremata and Thoracotremata (Ng et al., 2008; Tsang et al., 2014; Davie et al., 2015). The tree topology of superfamilies in these sections/subsections was complex; some parts of superfamilies were monophyletic—e.g., Majoidea, Portunoidea and Xanthoidea—and some were polyphyletic—e.g., Grapsoidea and Ocypodoidea (Tsang et al., 2014; Ma et al., 2019).

The habitats of different clades are diverse and include marine, intertidal, terrestrial and freshwater zones. Environmental factors in marine and subtidal zones—such as salinity (33–35 ppt), water content and temperature—yield no or slight fluctuations in status (Barnes, 1974; Little, 1990). Hyper-osmotic environment will cause the ion influxes and water loss stresses in organism (McNamara and Faria, 2012). Species in superfamilies such as Calappoidea, Dromioidea, Majoidea, Corystoidea, Goneplacoidea, Carpilioidea, Dorippoidea, Leucosioidea, Portunoidea, Pilumnoidea and Xanthoidea (belonging to Dromiacea and Heterotremata) are distributed in these habitats (Ng et al., 2008; Tsang et al., 2014; Davie et al., 2015; Naderllo, 2017). Crabs in Portunoidea, Pilumnoidea, Xanthoidea, Eriphioidae, Leucosioidea, Trapezioidea, Grapsoidea and Ocypodoidea (in clades Thoracotremata and Heterotremata) inhabit the intertidal zone (Takeda et al., 1996; Ng et al., 2008; Tsang et al., 2014; Shin et al., 2015; Naderllo, 2017), where there is a salinity gradient from marine to estuarine (about 5 ppt) and water content that fluctuates based on the daily tidal cycles (Barnes, 1974). Ion regulatory functions help the individual overcome dramatic salinity and water content changes over a short period (McNamara and Faria, 2012).

Species in terrestrial habitats showed a different way to maintain their water balance, gas exchange and ammonia excretion compared to the aquatic one (Little, 1990; Weihrauch et al., 2004). Terrestriality among crabs is categorized into five grades (grades of terrestriality; *T*-grades) (Hartnoll, 1988). Species in grade T3 and above are considered terrestrial (Li and Chiu, 2019); this includes Gecarcinidae (Grapsoidea); Ocypodidae (Ocypodoidea); and Potamoidea, Gecarcinucoidea and Eriphioidea (Thoracotremata and Heterotremata) (Ng et al., 2008; Davie et al., 2015; Shin et al., 2015; Naderllo, 2017; Li and Chiu, 2019). With the exception of primary freshwater crabs, terrestrial species migrate to coastal and intertidal areas during the breeding season to release their zoea (Saigusa, 1980). Potamoidea, Gecarcinucoidea, Pseudothelphusoidea and Trichodactyloidea (Heterotremata) invaded fully freshwater habitats, which have a low ionic concentration, on two occasions (Barnes, 1974; Ng et al., 2008; Lee et al., 2011; Tsang et al., 2014; Davie et al., 2015; Ma et al., 2019). Some freshwater species, for example, *Austrothelphusa transversa* which be called desert crab in Gecarcinucoidea, showed high terrestriality which inhabited in arid burrow during dry period (Taylor and Greenaway, 1979; Greenaway, 1984a). Diadromous species such as Varunidae (Grapsoidea) inhabit freshwater, but they migrate to estuaries through streams or rivers during the breeding season (Kobayashi, 1998; Ng et al., 2008; Tsang et al., 2014). Crabs can also inhabit extreme conditions. For example, species of Xenograpsidae (Grapsoidea) and Bythogaeidae (Bythograeoidea) live around hydrothermal vents, which have a low pH and high sulfide concentrations (Martinez et al., 2001; Hu et al., 2016; Allen et al., 2020).

## Brief Introduction to Osmoregulatory Patterns in Brachyurans

Species in various environments have different osmoregulatory and ion regulatory patterns. They exhibit variation between being an osmo-conformer and an osmoregulator (Péqueux, 1995; Charmantier et al., 2009). The hemolymph osmolality of osmo-conformers changes following the isosmotic line between hemolymph and environment, but they can still regulate their cell volume and be an ion-regulator which modify their hemolymph ion composition (McNamara and Faria, 2012). Most of the marine (deep sea) crustaceans are considered osmo-conformers (McNamara and Faria, 2012). The osmoregulators regulate their hemolymph osmolality by actively absorbing or secreting ions. This group can be further divided into two types: the hyper-osmoregulators and the hyper-hypo-osmoregulators (Péqueux, 1995; Charmantier et al., 2009). Hyper-osmoregulators can sustain a higher hemolymph osmolality than that of their environment up to the isosmotic point, but they will become an osmo-conformer in hyper-osmotic mediums, such as freshwater species and some intertidal species (Shaw, 1958; Gross, 1964; Chung and Lin, 2006; Charmantier et al., 2009). The hyper-/hypo-osmoregulators can keep their hemolymph osmolality within a limited range, regardless of the environmental osmolality; for example, *Minuca* and *Leptuca* species can maintain their hemolymph osmotic concentration lower and higher than the isosmotic line between hemolymph and environment in hyper-osmotic and hypo-osmotic environments, respectively (Thurman, 2005; Faria et al., 2017).

The underlying mechanism of different osmoregulatory patterns among crabs are promoting by two important active transporters—Na^+^, K^+^-ATPase (NKA) and V-type H^+^-ATPase (VHA)—which are the crucial driving forces behind the ion regulatory process (Brown and Breton, 1996; Kirschner, 2004). NKA, a P-type ATPase, is an important enzyme that transports three Na^+^ ions into the hemolymph and brings two K^+^ ions into the cytoplasm to generate a Na^+^ and K^+^ concentration gradient and trans-membrane potential difference (Lodish et al., 2000; Jorgensen et al., 2003). NKA is composed of *α*, *β*, and *γ* subunits; the *α* subunit—the largest one—was the site ion transport process occurs (Jorgensen et al., 2003; Sáez et al., 2009). Protein kinase A (PKA), protein kinase C (PKC) and Ca^2+^/calmodulin-dependent kinase (CaMK) can inhibit the gill NKA activity of *Ucides cordatus* by phosphorylation (Leone et al., 2020). *β* and *γ* subunits interacted with *α* subunit to stable *α* subunit structure (Jorgensen et al., 2003; Sáez et al., 2009) or interacted with other proteins, such as FXYD, to regulate the activity of NKA (Jorgensen et al., 2003). For example, the gill NKA activity of *U. cordatus* increased about 1.8 fold in low salinity condition when exogenous FXYD2 was present (Leone et al., 2020). NKA will prompt the Na^+^ and Cl^−^ absorption mechanism in gills of crabs during hypo-osmotic stress with other ion transporters. Na^+^, K^+^, 2Cl^-^, symporter (NKCC) transport Na^+^ and Cl^−^ by electrochemical gradient generate by basolateral NKA, Cl^−^ channel, K^+^ channel and apical K^+^ channel; Na^+^, H^+^, exchanger (NHE) exchanges Na^+^ by Na^+^ gradient produce by NKA and H^+^ gradient by carbonic anhydrase (CA)—can catalyze the H_2_O and CO_2_ to H^+^ and HCO_3_^−^ and vice versa—in an osmo-conformer or weak hyper-osmoregulator (Kirschner, 2004; Freire et al., 2008; Henry et al., 2012; McNamara and Faria, 2012; Allen and Weihrauch, 2021). In addition, Cl^−^ absorption will also be executed by Cl^−^, HCO_3_^−^, exchanger (AE), that is, driven by the HCO_3_^−^ gradient which is produced by CA (Kirschner, 2004; Freire et al., 2008; Henry et al., 2012; McNamara and Faria, 2012; Allen and Weihrauch, 2021). On the other hand, NKA, basolateral NKCC and apical Cl^−^ channel involved in the Na^+^ and Cl^−^ secretion in gills when crabs were subjected to hyper-osmotic condition (Freire et al., 2008; Henry et al., 2012; McNamara and Faria, 2012).

VHA, considered an acid-base regulatory enzyme, also involved in the ion regulatory process (Klein, 1992; Onken and Putzenlechner, 1995; Beyenbach and Wieczorek, 2006). VHA in the intercalated cells (ICs) of the rat kidney and in the Madin-Darby Canine Kidney cell line (MDCK cell) participates in ion regulation (Feifel et al., 1997; Silver et al., 2000). VHA is also believed to participate in ion regulation in the Malpighian tubules and the gut goblet cells of insects, frog skin, crustacean gill and fish gill (Klein, 1992; Maddrell and O’Donnell, 1992; Ehrenfeld and Klein, 1997; Wieczorek et al., 1999; Jensen et al., 2002). In brachyuran, *Carcinus maenas*, a weak hyper-osmoregulators, VHA distributed in cytoplasm of gills and the transepithelial potential was not affected by bafilomycin, a VHA inhibitor (Weihrauch et al., 2001). In addition, VHA mRNA expression was higher in the anterior gills than the posterior ones in hypo- and hyper-osmotic conditions (Weihrauch et al., 2001). These evidences indicated that VHA in gills of *C. maenas* might majorly involves in the organelle acidification but not in Na^+^ or Cl^−^ absorption (Weihrauch et al., 2001). In contrast, VHA involves in ion regulation of strong hyper-osmoregulators in hypo-osmotic condition. The short-circuit current or transepithelial potential difference in gills of *Eriocheir sinensis* and *Chasmagnathus granulatus* were reduced when the bafilomycin was present in the apical sides of epithelium (Onken and Putzenlechner, 1995; GenoveseOrtiz et al., 2005). This indicated that the VHA in gills of these two species was involved in Cl^−^ absorption (Onken and Putzenlechner, 1995; GenoveseOrtiz et al., 2005). In addition, the gill VHA mRNA expression of freshwater species, *Dilocarcinus pagei*, decreased when species was subjected to the hyper-osmotic stress for reducing the V-ATPase-dependent ions absorption (Mantovani and McNamara, 2021). A hypothesis was proposed that the apically located VHA is for excreting H^+^ directly in acid-base regulation or generating the electric gradient for ion regulation; while the cytoplasmic one is for organelle acidification that can further secrete the proton or produce “acid-trapping” to transport the ammonia into vesicle and secrete from apical membrane of gills by exocytosis (Wieczorek et al., 1999; Weihrauch et al., 2001; Jensen et al., 2002; Kirschner, 2004; Weihrauch et al., 2004; Hu et al., 2016).

Crab osmoregulatory patterns are supported by the distinct arrangement of proteins in ion regulatory organs, such as gills and antennal glands. But even species with similar osmoregulatory patterns and habitats can also have completely different strategies to sustain their homeostasis. Physiological plasticity is an important mechanism for some species to at least temporarily maintain their homeostasis in multiple habitat types (Henry, 1994). Local adaptation may occur when populations of the same species invade to the different habitat types with different environmental stress (Kawecki and Ebert, 2004). This phenomenon may be one of a possible process for speciation (Savolainen et al., 2013). Studies and reviews showed that the structure and ion regulatory functions of gills differ among crabs in different habitats and lineages (Takeda et al., 1996; Tsai and Lin, 2007; Freire et al., 2008; Charmantier et al., 2009; Henry et al., 2012; McNamara and Faria, 2012; Allen and Weihrauch, 2021). This phenomenon may correlate with ion regulation in the brachyuran antennal gland; we will summarize the evidence for this in later sections.

## Comparison of Structure and Ion Regulation in the Antennal Gland Among Crabs

The antennal gland—which is functionally similar to the kidney of vertebrates and the Malpighian tubule of insects—is believed to be the excretory organ that plays an important role in the volume and ion composition of hemolymph regulation (Feifel et al., 1997; Lin et al., 2000; Freire et al., 2008; Brown et al., 2009). The water turnover of an individual can be regulated by different urine production rates in crabs among various environments. Researchers used the clearance rate of isotope material, such as inulin with ^14^C or EDTA with ^51^Cr, to estimate the urine production rate of crabs in different mediums (Riegel and Lockwood, 1961; Binns, 1969; Kormanik and Harris, 1981; Morris and Ahern, 2003). For example, the intertidal species *Carcinus maenas* and shore crab *Pachygrapsus crassipes* can increase their urine production rate when salinity decreases (Gross and Marshall, 1960; Binns, 1969). The urine flow and EDTA clearance of the land crab *Gecarcoidea natalis* during the wet season was higher than that during the dry season (Morris and Ahern, 2003). *Gecarcinus lateralis* showed a higher urine production rate when it came in contact with the moist sand compared to the dry sand (Harris, 1977). Most brachyuran species can only produce isosmotic urine, but some of them can modify the ion composition of their urine to distinguish it from hemolymph (Gross, 1964; Kormanik and Harris, 1981; Weihrauch et al., 2004; Tseng et al., 2020). The intertidal species *Tubuca arcuata* produces an isosmotic urine in 5 and 35 PSU seawater, but its urine-hemolymph ratio (U/B) for Na^+^ and Cl^−^ is lower and higher than 1, respectively. These results indicated that *T. arcuata* could reabsorb Na^+^ and excrete Cl^−^ from urine in different environments (Tseng et al., 2020).

### Structure and Distribution of Ion Regulatory Proteins in the Antennal Gland

The structure and distribution of ion regulatory proteins in the antennal gland of crayfish are well-documented and reviewed (Peterson and Loizzi, 1974a; Roldan and Shivers, 1987; Wheatly and Henry, 1987; Sarver et al., 1994; Wheatly and Gannon, 1995; Ueno and Inoue, 1996; Khodabandeh et al., 2005; Freire et al., 2008; Charmantier et al., 2009). The antennal gland of crayfish contains three parts: the coelomosac, labyrinth and nephridial canal (Peterson and Loizzi, 1973; Peterson and Loizzi, 1974a; Freire et al., 2008; Charmantier et al., 2009). The coelomosac is composed of the coelomic cells connected to adjacent cells by foot processes similar to those of the podocytes. It is considered the place to produce primary urine (Peterson and Loizzi, 1973; Peterson and Loizzi, 1974a; Ueno and Inoue, 1996). The labyrinth contains cells with apical microvilli, membrane folding, and mitochondria and it is analogous to the proximal tubule in the vertebrate kidney (Peterson and Loizzi, 1973; Peterson and Loizzi, 1974a; Roldan and Shivers, 1987). Finally, the nephridial canal—which has thicker cells and higher levels of Mg^2+^-dependent ATPase than other parts of the antennal gland—is functionally similar to the distal tubule (Peterson and Loizzi, 1973; Peterson and Loizzi, 1974a). Peterson and Loizzi (1974b) found that the labyrinth and nephridial canal of *Procambarus* species had a higher level of NKA activity and distribution than the coelomosac. Khodabandeh et al. (2005) also found a similar result in the antennal gland in embryos and juveniles of the crayfish *Astacus leptodactylus*. In addition, the urine chloride concentration and osmotic pressures were lower in the distal part of nephridial canal of *Austropotamobius pallipes* than in the coelomosac and labyrinth (Riegel, 1963). These evidences may indicate that expression level and activity of NKA were correlated to the ionic and osmotic regulation ability and the distal part of nephridial canal with higher level of NKA activity and distribution was a presumed major site for ion regulation in antennal gland of crayfish (Charmantier et al., 2009).

**FIGURE 1:**
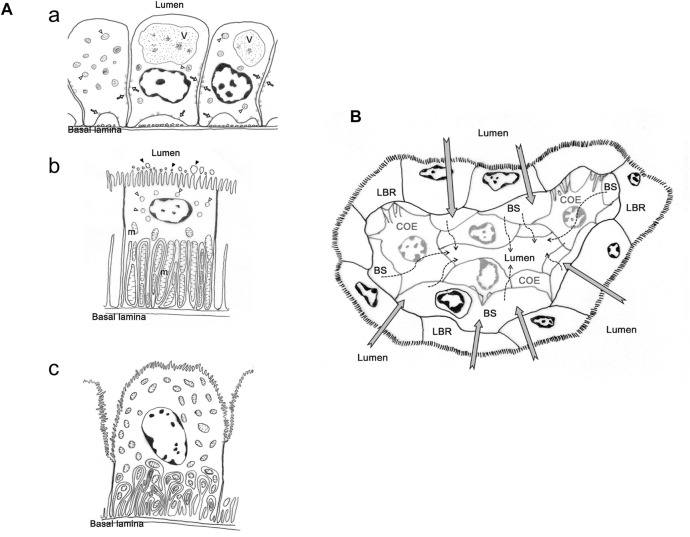
The cell types and presumed urine production and re-absorption process in antennal gland of *Ocypode stimpsoni*. **(A)** Three types of cell in antennal gland of *O. stimpsoni*. Coelomic cells with different size of vesicle (open arrow and triangle) and endosome (V), **(a)**. Labyrinthine cells have a great of mitochondria (m) in basal folding, vesicles (open triangle) in sub-apical region and aposomes (black triangle) around apical microvilli, **(b)**. End-labyrinthine cells, another type of labyrinthine cells, showed a different shape of apical membrane compared to labyrinthine cells. In addition, mitochondria not only distributed in basal folding, but also in other regions of end-labyrinthine cells, **(c)**. **(B)** Hemolymph will be filtrated through coelomic cells (dash arrow), and the ions and other substances will be re-absorbed into hemolymph by labyrinthine cells (grey arrow). COE, coelomic cell. hs, haemolymph sinus. LBR, labyrinthine cell. The figure comes from Tsai and Lin, (2014).

In contrast to crayfish, studies on the structure and distribution of ion regulatory proteins in the antennal gland of brachyuran crabs are limited to the genera *Uca*, *Ocypode* and *Callinectes* (Schmidt-Nielsen et al., 1968; Johnson, 1980; Tsai and Lin, 2014). Two major cell types, coelomic and labyrinthine cells, were discovered in the antennal gland of crabs (Schmidt-Nielsen et al., 1968; Johnson, 1980; Tsai and Lin, 2014) (Figure 1A). McGaw (2005) used a perfusion method to construct the cardiovascular system of *Cancer* species. The study showed that the coelomosac artery (CCA) extends into the antennal gland and becomes branches that form circular structures; the empty space in the circle is considered to be where the coelomosac and labyrinth originally resided (McGaw, 2005). The coelomosac is surrounded by the labyrinth, and hemolymph sinus is between these two structures (Schmidt-Nielsen et al., 1968; Johnson, 1980). Based on the cardiovascular path and ultrastructure of the antennal gland, Tsai and Lin (2014) suggested that primary urine is produced by the foot processes of the coelomosac connected with the capillaries from CCA—urine flows into the lumen, where ions are reabsorbed by the labyrinth back into the capillaries (Figure 1B).

However, there were several different features in ultrastructure among labyrinthine cells in different part of antennal gland (Schmidt-Nielsen et al., 1968; Tsai and Lin, 2014). Schmidt-Nielsen et al. (1968) found at least two types of cells in the labyrinth, one with more mitochondria and basolateral infolding, but these structures were fewer in other cell type in *M. mordax*. In *O. stimpsoni*, the thickness and length of apical microvilli in labyrinthine cells are different between proximal and distal tubular regions in the head part of the antennal gland. In addition, the so-called end-labyrinthine cell (ELBR) in the tail part of the antennal gland is even thicker and the cell’s apical microvilli are even longer than in the head part of the antennal gland (Tsai and Lin, 2014) (Figure 1A).

McNamara et al. (2015) compared the density of apical microvilli and the basal invaginations of the antennal gland among six palaemonid shrimps from intertidal to freshwater zones in *Macrobrachium* and *Palaemon*. The species in *Macrobrachium* in the freshwater habitat mainly had higher apical microvilli and basal invagination densities in the antennal gland; this lineage also showed a higher gill apical evaginations density. None of this was the case for *Palaemon* in the intertidal zone. (McNamara et al., 2015). In addition, the osmotic gradient between hemolymph and environment was greater in *Macrobrachium* species. The authors indicated that the extension of surface area in the gills and antennal gland of *Macrobrachium* species can facilitate the ion and water regulation in freshwater environment. This phenomenon may be caused by natural selection in these species (McNamara et al., 2015). However, more data on the structure of the antennal gland are needed among crabs to do meta-analyses for comparing the structural differences among species in different habitats and phylogenetic clades.

Furthermore, the arrangement and distribution level of ion regulatory proteins in the antennal gland are also different among coelomic, labyrinthine and end-labyrinthine cells (Tsai and Lin, 2014). NKA distributed in the basolateral regions of coelomic and two types of labyrinthine cells—VHA and NKCC—were found in the vesicles and apical region of the coelomic and labyrinthine cells, respectively. The intensity of the immunocytochemical stain in NKA is highest in ELBR, and coelomic cell only showed a slight NKA distribution (Tsai and Lin, 2014). This result is similar to the NKA distribution in the antennal gland of crayfish (Peterson and Loizzi, 1973; Khodabandeh et al., 2005). The labyrinthine cells showed various ultrastructures and ion regulatory protein distribution in different parts of the antennal gland in *Minuca mordax* and *Ocypode stimpsoni*, but the linkage between the structure and functions of labyrinthine cells needs further investigation.

Previous research proposed a possible pathway by which various ion regulatory protein arrangements in gills evolved among brachyurans based on phylogenetic relationships and ion regulatory patterns (McNarmara and Faria, 2012). The presumed ancestral status is similar to osmo-conformers in marine species; the apical NHE losses and VHA are independent in strong hyper-osmoregulators in freshwater and diadromous clades; and apical and the basal NKCC, basal K^+^ channel and basal Cl^−^ channel are found in weak hyper-osmoregulators in brackish water, as well as semi-terrestrial and diadromous species (McNarmara and Faria, 2012). If the evolution of ion regulatory protein arrangements in the antennal gland can be further investigated, the possible process that how the evolution and environment shape the function of antennal gland among brachyuran may also be speculated.

### Differences in Ion Regulatory Functions of Antennal Glands Among Crabs

**FIGURE 2:**
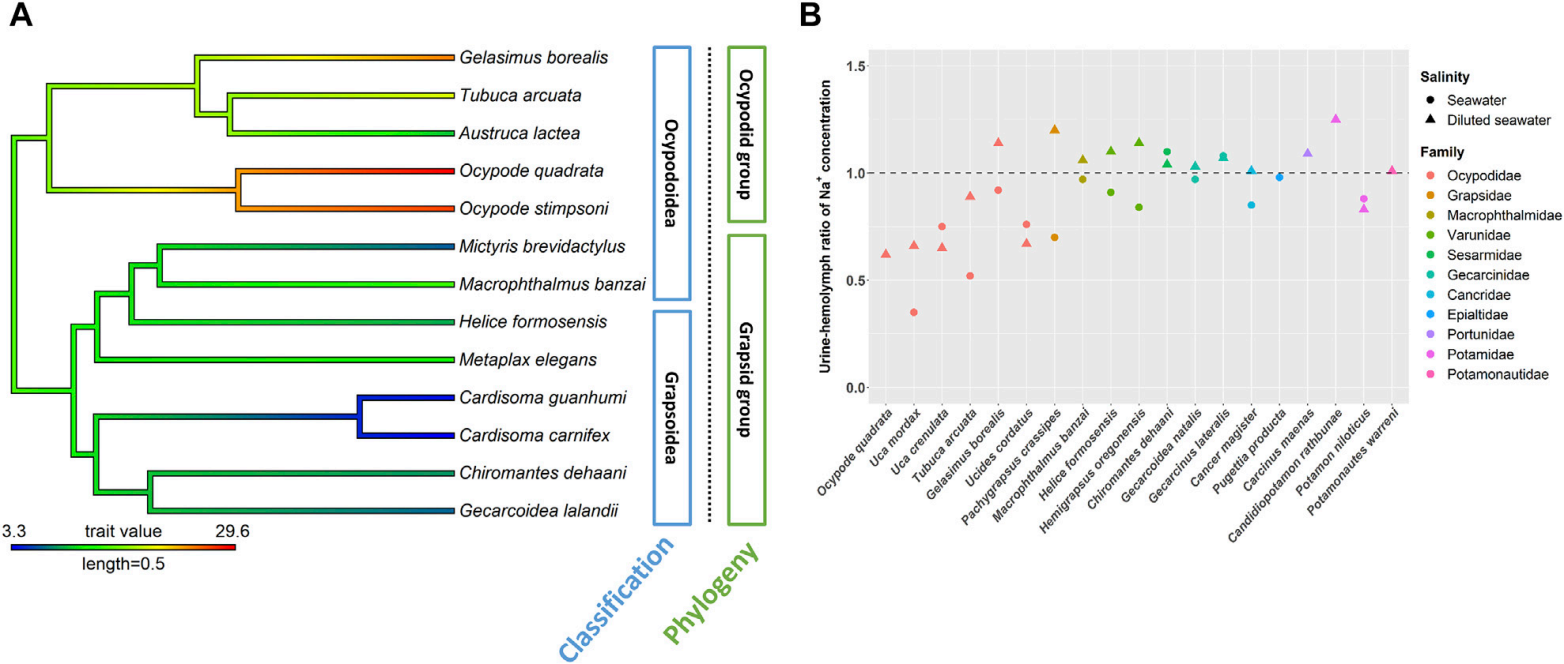
The NKA activity and urine and hemolymph ratio (U/H) for Na^+^ in the antennal glands of different crab species. The phylogenetic tree was generated by Bayesian inference with COI and 16S rDNA. The antennal gland NKA activity of crabs in 5 ppt was mapped on the phylogenetic tree and the ancestor status was predicted by the phylogenetic structure. Labels in blue boxes were the superfamilies of brachyuran. Labels in green boxes were the groups separated by the clades in phylogenetic trees. **(A)** Antennal gland NKA activity in the ocypodid group was higher than that in the grapsid group. **(B)** Ocypodidae species had a lower U/H for Na^+^ than did species in other families. The figure comes from Tseng et al. (2020).

The involvement of ion regulatory functions in the antennal gland varied among crab species (Gross, 1964; Bliss, 1968; Harris and Santos, 1993; DeVries et al., 1994; Morris, 2001; Weihrauch et al., 2004; Freire et al., 2008; Charmantier et al., 2009; Tseng et al., 2020) (Figure 2). Among Ocypodidae species, *Ocypode quadrata* had high terrestriality and showed an ability to reabsorb Na^+^ and excrete NH_4_^+^ from urine during hypo-osmotic stress (DeVries et al., 1994). This phenomenon also occurred in some *Uca* and Gelasiminae species. *Minuca pugnax* can reabsorb Na^+^ and excrete NH_4_^+^ from urine in 100 and 175% seawater (Green et al., 1959). The Na^+^ concentration in the urine of the semi-terrestrial species *Lepuca crenulata* was lower than that in the hemolymph in 50, 100 and 150% seawater (Gross, 1964). The semi-terrestrial species *Minuca mordax* and bimodal/intertidal species *Tubuca arcuata* can reabsorb Na^+^ from urine in both hypo-osmotic and hyper-osmotic mediums (Schmidt-Nielsen et al., 1968; Tseng et al., 2020). In addition, the bimodal/intertidal species *Ucides cordatus* has a relatively low urine to hemolymph ratio (U/H) with Na^+^ concentration in diluted seawater (Harris and Santos, 1993). On the other hand, the following only showed a limited ability to reabsorb Na^+^ from urine in hypo-osmotic mediums: the terrestrial species *Gecarcoidea natalis* and *G. lateralis* (Gecarcinidae) (Gross, 1964; Taylor and Greenaway, 2002); semi-terrestrial/intertidal crabs *Chiromantes dehaani* (Sesarmidae), *Helice formosensis* and *Hemigrapsus oregoncnsis* (Varunidae) (Gross, 1964; Tseng et al., 2020; Allen and Weihrauch, 2021); intertidal/marine species *Cancer antennarius* and *Cancer magister* (Cancridae) (Gross, 1964; Hunter and Rudy, 1975), *Carcinus maenas* and *Callinectes sapidus* (Portunidae) (Carmeron and Batterton, 1978; Harris and Santos, 1993) and *Macrophthalmus banzai* (Macrophthalmidae) (Tseng et al., 2020); and freshwater species *Candidiopotamon rathbunae* (Wang and Lin, 2011) and *Potamonautes warreni* (Morris and Van Aardt, 1998) (Figure 2B). Two Varunidae species, *Pachygrapsus crassipes* (Grapsidae) and two Cancridae species can reabsorb Na^+^ from urine to some degree in hyper-osmotic environments (Prosser et al., 1955; Gross, 1964; Hunter and Rudy, 1975; Harris and Santos, 1993; Tseng et al., 2020; Allen Weihrauch, 2021). Although crabs showed different degrees of magnesium excretion from urine, species of Cancridae, Gecarcinidae, Grapsidae, Ocypodidae, Varunidae and Grapsidae can excrete/lose Mg^2+^ from urine in hypo- and/or hyper-osmotic environments (Gross, 1964; Hunter and Rudy, 1975; DeVries et al., 1994; Taylor and Greenaway, 2002; Allen and Weihrauch, 2021).

**FIGURE 3:**
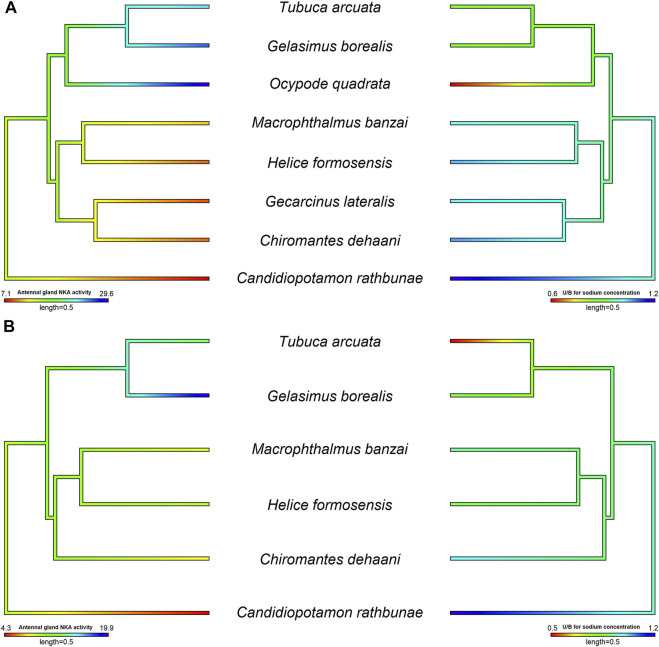
The relationship between antennal gland NKA activity and U/H for Na^+^ concentration in the antennal gland of different crab species in hypo- and hyper-osmotic environments. The phylogenetic tree was generated by Bayesian inference with COI, 12S and 16S rDNA. The antennal gland NKA activity and U/H for Na^+^ concentration of crabs in 5 or 35 ppt were mapped on the phylogenetic tree and the ancestor status was predicted by the phylogenetic structure. The correlation between antennal gland NKA activity and U/H for Na^+^ concentration was analyzed by PGLS. Left pictures were the results of antennal gland NKA activity, and right sides were U/H for Na^+^ concentration. **(A)** The antennal gland NKA activity was correlated with the U/H for Na^+^ concentration in the antennal gland when species were transferred into hypo-osmotic environments (*t* value = −5.7963, df = 8, *p* = 0.0012, R^2^ = 0.88). **(B)** There was no correlation between antennal gland NKA activity and the U/H for Na^+^ concentration in the antennal gland when species were transferred into hyper-osmotic mediums (*t* value = −0.1627, df = 6, *p* = 0.8786, R^2^ < 0.001). Data of *Ocypode quadrata* are from DeVries et al. (1994). Data of *Gecarcinus lateralis* are from Gross (1964) and DeVries et al. (1994). Data of *Candidiopotamon rathbunae* are from Wang and Lin (2011).

The Na^+^, K^+^-ATPase (NKA) activity in the antennal gland showed a similar pattern to the U/H for Na^+^ concentration among crabs in hypo-osmotic environments (DeVries et al., 1994, Weihrauch et al., 2004; Tsai and Lin, 2014; Tseng et al., 2020) (Figures 2, 3). *Ocypode stimpsoni*, *O. quadrata* and two Gelasiminae species had a relatively higher antennal gland NKA activity in hypo-osmotic environments (DeVries et al., 1994; Tsai and Lin, 2014; Tseng et al., 2020). In contrast, *Austruca lactea*, *Cardisoma carnifex*, *Cardisoma guanhumi*, *Chiromantes dehaani*, *Gecarcoidea lalandii*, *Helice formosensis*, *Macrophthalmus banzai*, *Metaplax elegans*, *Mictyris brevidactylus*, *Scylla paramamosain* and *Ucides cordatus* exhibited a lower antennal gland NKA activity in diluted mediums (Schmidt-Nielsen et al., 1968; Towle, 1981; Harris and Santos, 1993; DeVries et al., 1994; Chung and Lin, 2006; Tseng et al., 2020). Based on this, habitat type, which is considered an important factor controlling the diverse physiological patterns of crabs, seems to not be the only factor driving the evolution of this distinct ion regulation mechanism in the antennal gland.

Tsang et al. (2014) indicated that, in Ocypodoidea and Grapsoidea, some of the families in one superfamily are more closely related to a family in the other—e.g., Macrophthalmidae and Mictyridae (Ocypodoidea) are more closely related to families in Grapsoidea—such as Varunidae—than the other Ocypodidae in the same superfamily. This finding implies an underlying possibility that phylogeny affects the physiological patterns of the antennal gland among crabs. Tseng et al. (2020) compared the antennal gland NKA activity among Ocypodoidea and Grapsoidea in a hypo-osmotic environment by phylogenetic ANOVA and Moran’s *I* auto-correlation. The results showed that the antennal gland NKA activity of crabs is phylogenetically correlated and significantly different between ocypodid and grapsid groups (Tseng et al., 2020) (Figure 2A). In the present review, we further used phylogenetic generalized least square (PGLS) to analyze whether there is a correlation between antennal gland NKA activity and U/B for Na^+^ concentration in hypo-osmotic environments among crabs. The result showed that these two features were correlated (*t* value = -5.7963, df = 8, *p* = 0.0012, R^2^ = 0.88). Species with higher antennal gland NKA activity showed a lower U/B for Na^+^ concentration under hypo-osmotic stress (Figure 3A). However, *Ocypode* species showed a greater antennal gland NKA activity and Na^+^ reabsorption capacity than did Gelasiminae and *Uca* species. This is an indication that the terrestriality of crabs still plays an important role in the ion regulatory functions of the antennal gland in Ocypodidae species (DeVries et al., 1994; Tsai and Lin, 2014; Tseng et al., 2020).

On the other hand, crabs’ capacities to reabsorb Na^+^ from urine under hyper-osmotic environments had a similar pattern to the capacities under hypo-osmotic stress, Ocypodidae species showed a higher Na^+^ reabsorption ability from urine (Figure 2B). However, the antennal gland NKA activity of *Tubuca arcuata*—which contained a low U/H for Na^+^ concentration—did not differ from those of *Helice formosensis* and *Macrophthalmus banzai*—which rarely or slightly reabsorb Na^+^ from urine—in a 100% seawater environment (Tseng et al., 2020) (Figure 3B). Based on the PGLS analysis, Na^+^ reabsorption from urine did not correlate to the antennal gland NKA activity among crabs in hyper-osmotic mediums (*t* value = -0.1627, df = 6, *p* = 0.8786, R^2^ < 0.001). On the other hand, the antennal gland is also involved in Mg^2+^ regulation in brachyurans. Although the trend in U/H for magnesium (Mg^2+^) concentration among crabs corresponds to a trend in Na^+^ reabsorption capacity from urine, the U/H for Mg^2+^ concentration of Cancridae, Gecarcinidae, Grapsidae, Ocypodidae, Varunidae and Grapsidae species was >1 in hypo- and/or hyper-osmotic environments (Green et al., 1959; Gross, 1964; Taylor and Greenaway, 2002). Green et al. (1959) indicated that Na^+^ reabsorption and Mg^2+^ excretion from urine may be correlated; for example, *P. crassipes*, *L. crenulata*, *L. pugilator*, *M. pugnax* and *H. oregonensis* had a U/H for Na^+^ concentration < 1 and a U/H for Mg^2+^ concentration >1 in seawater (Prosser et al., 1955; Green et al., 1959; Gross, 1964). In addition, the U/H for the NH_4_^+^, SO_4_^2-^ and Ca^2+^ concentrations of *L. crenulata* and *L. pugilator* were >1 in 100 and 175% seawater (Green et al., 1959). However, in *G. lateralis*, *G. natalis* and *C. antennarius*, the U/H for Na^+^ concentration was close to one and the Mg^2+^ concentration in urine was higher than that in the hemolymph (Gross, 1964; Taylor and Greenaway, 2002). The relationship between Na^+^, NH_4_^+^ and divalent ion regulation in hyper-osmotic environments and which ion regulatory proteins are involved in these mechanisms in antennal glands need further investigation.

One reason why ion regulation in the antennal gland differs among crabs may be correlated with the differences in the morphology and function of gills and lung-like structures in different brachyuran clades. Takeda et al. (1996) indicated that land crabs in Ocypodoidea and Grapsoidea invaded terrestrial habitats through different routes: Ocypodid species directly from intertidal/supratidal zones and Grapsid species through estuaries and rivers/streams. The terrestrial species *Gecarcinus lateralis* in Gecarcinidae and *Chiromantes dehaani* in Sesarmidae have eight or nine pairs of gills, similar to marine, intertidal and bimodal species such as *Macrophthalmus banzai* in Macrophthalmidae, *Scylla paramamosain* in Portunidae and *Helice formosensis* and *Metaplax elegans* in Varunidae, which also had eight pairs of gills (Copeland and Fitzjarrell, 1968; Takeda et al., 1996; Tseng et al., 2020). However, Ocypodidae species—such as the semi-terrestrial species *Ocypode stimpsoni*; intertidal species *Austruca lactea*; and intertidal/bimodal species *Gelasimus borealis*, *Tubuca arcuata*, and *Xeruca formosensis*—have an inconsistent reduced number of pairs of gills (Takeda et al., 1996; Lin et al., 2002; Tsai and Lin, 2012; Tseng et al., 2020).

In addition, the lung types and properties were also different among crabs and the degree of complexity was higher in Ocypodidae than in Grapsidae. The lung-like structure was specialized from the branchiostegite and formed a complex folding with branching hemolymph vessels in the inner side of the branchial chamber (Greenaway, 1984b; Farrelly and Greenaway, 1987; 1993; Greenaway and Farrelly, 1990; Greenaway, 1999). *Ocypode* species had a highly compact and evaginated lung; in contrast, Gecarcinidae, Grapsidae and Varunidae species showed expanded and smooth lungs (Tsai and Lin, 2012) (Table 1). Investigations suggested the lung can increase the efficiency of gas exchange when it is active on land (Taylor and Greenaway, 1979; Innes and Taylor, 1986).

**TABLE 1:** Lung types and habitats of crabs among families.

Superfamily	Family	Habitat^†^	Species	Lung type^‡^			References
				Cp/Ex	Sm/Ev/Iv/Un	2D/3D	
Ocypodoidea	Ocypodidae	T	*Ocypode ceratophthalmus*	Cp	Ev	3D	(3)
		T	*Ocypode cordimanus*	Cp	Ev	3D	(1)
		T	*Ocypode stimpsoni*	Cp	Ev	3D	(5)
		UT	*Tubuca coarctata*	Cp	Sm	2D	(3)
	Mictyridae	IT	*Mictyris longicarpus*	Cp	Iv	3D	(1), (3)
Grapsoidea	Grapsidae	UT	*Hemigrapsus nudus*	Ex	Sm	2D	(2)
		T	*Geograpsus grayi*	Ex	Sm	2D	(3)
	Gecarcinidae	T	*Cardisoma hirtipes*	Ex	Sm	2D	(3)
		T	*Cardisoma carnifex*	Ex	Sm	2D	(3)
		T	*Gecarcoidea natalis*	Ex	Sm	2D	(2)
	Varunidae	U	*Neohelice granulatus*	Ex	Sm	2D	(4)
Gecarcinucoidea	Gecarcinucidae	Fw/T	*Austrothelphusa transversa*	--	Sm	--	(2), (3)

^†^Habitat: T, terrestrial; IT, intertidal; UT, upper intertidal; Fw, freshwater.

^‡^Lung type: Cp, compact; Ex, expanded; Sm, smooth; Ev, evaginated; Iv, invaginated; Un, unclear lung type, indicating that no high quality data was obtained by paraffin section 2D: the lung was extended through a 2-dimensional direction. 3D, the lung was extended through a 3-dimensional direction. --, data was not available.

References: (1) Farrelly and Greenaway, 1987. (2) Greenaway and Farrelly, 1990. (3) Farrelly and Greenaway, 1993. (4) Halperin et al., 2000. (5) Tsai and Lin, 2012. Table was modified from Tsai and Lin, 2012.

The development of the lung from the branchiostegite may cause a functional shift in gas exchange from the gill to lung; in addition, the principal site for gas exchange—the anterior gills—becomes involved in ion regulation (Innes and Taylor, 1986; Santos et al., 1987; Greenaway and Farrelly, 1990; Tsai and Lin, 2012). *Tubuca arcuata*, *Austruca annulipes*, *Tubuca urvillei*, *Gelasimus tetragonon* and *Xeruca formosensis* have reduced pairs of gills and an osmoregulatory lamella in their anterior gills (Takeda et al., 1996; Lin et al., 2002). The NKA activity of the anterior gills in *Ocypode stimpsoni* significantly increased 4 days after being transferred into 5 ppt seawater (Tsai and Lin, 2012). In these species, *Ocypode* spp. and *Tubuca arcuata* also showed a relatively high NKA activity in the antennal gland and Na^+^ absorption ability from urine (DeVries et al., 1994; Tsai and Lin, 2014; Tseng et al., 2020) (Figure 3A). The osmoregulatory functional shifts may not only occur in anterior gills, but also to the antennal gland (Tsai and Lin, 2012; Tsai and Lin, 2014). This phenomenon may lead to differences among species in ion regulation in the gills and antennal gland. However, more studies are needed to fill the missing data in other phylogenetic clades of brachyurans. This will lead to a more comprehensive phylogeny of ion regulatory functions in brachyurans.

## Multi-Omics Methods for Ion Regulation Studies in Crabs

Integrating other physiological features to describe the ion regulatory functions in organisms can let us compare the ion regulatory difference among species in a comprehensive way. Next generation sequencing (NGS) generates a great deal of sequencing data in a short time; these data can then connect the physiological studies to multi-gene or genome level perspectives. Over the past decade, this technique has emerged as a mature method for facilitating the development of a wide range of disciplines (Schuster, 2008; Shendure and Ji, 2008; Liu et al., 2012; Goodwin et al., 2016). RNA sequencing by NGS (RNAseq) can simultaneously detect the expression level and number of functional genes to analyze the relationships between genes and physiological pathways in different environments (Wang et al., 2009). Proteomics method is another powerful tool to detect the expression of several proteins or discover novel proteins involve in different physiological pathways (Aslam et al., 2017; Suhre et al., 2020).

Researchers have used RNAseq to investigate the transcriptomes of organs in marine and freshwater crabs, most of them focusing on ion-regulatory gills (Lv et al., 2013; Li et al., 2014; Yang et al., 2019; Niu et al., 2020; Malik and Kim, 2021). Their results identified not only the routine responses of ion regulatory proteins, but other important physiological pathways, such as metabolism, signal transduction and anti-oxidation up-/down- regulation during salinity stresses in posterior gills (Lv et al., 2013; Li et al., 2014; Havird et al., 2016; Yang et al., 2019; Niu et al., 2020; Malik and Kim, 2021). For example, NKA and VHA was found to be downregulated and NKCC upregulated in *E. sinensis* in a seawater treatment; in addition, the expression of Cu^2+^/Zn^2+^ superoxide dismutase in the antioxidant activity pathway increased about 6.5 fold in a 25 ppt treatment (Yang et al., 2019). VHA and CA in ion regulation and Acetyl-CoA acetyltransferase in the metabolism pathway and signaling mucin HKR1 in the signal transduction pathway increased when *S. paramamosain* was transferred into a 5 ppt medium (Niu et al., 2020). These important physiological pathways can help individuals resist stress and maintain an energy homeostasis when salinity changes (Li et al., 2014; Yang et al., 2019; Niu et al., 2020).

Proteomics level changes of gills among salinity was also investigated in marine species (Wang et al., 2018; Niu et al., 2020). Wang et al. (2018) used SDS-PAGE and HPLC-MS to detect the proteome in gills of *Scylla paramamosain* under hypo-osmotic condition. KEGG pathway analysis results showed that proteins in amino acid metabolism and NKA regulation upregulated in 3 ppt seawater (Wang et al., 2018). These physiological pathways were considered as important mechanisms for osmotic and ionic regulation of brachyurans (Wang et al., 2018). In addition, Niu et al. (2020) showed that VHA subunit B and CA2 in gills of *S. paramamosain* not only upregulated in gene level, but also increased in protein level under hypo-osmotic stress. The protein expression of Cl^−^ channel, Rh protein for ion regulation and NADH dehydrogenase in energy metabolism of gills also increased in 5 ppt seawater (Niu et al., 2020). As the number of transcriptomic and proteomics studies increase and are applied to other brachyurans in intertidal or terrestrial habitats and different phylogenetic clades, we will become better equipped to compare the physiological responses of ion regulation.

On the other hand, Havird et al. (2016) used RNAseq to show that the gene expression levels between anterior and posterior gills differed in *Callinects sapidus* under different salinity stresses. The expressions of the NKA, NHE and Na^+^ channels in posterior gills were higher than in anterior ones at 35 ppt (Havird et al., 2016). And NKA expression in the posterior gills was also higher than in the anterior gills in the 5 ppt treatment (Havird et al., 2016). Furthermore, genes expression in the metabolism pathway also increase in the posterior gills under hypo-osmotic stress (Havird et al., 2016). Moshtaghi et al. (2016) analyzed the gene expressions in gills, antennal gland and hepatopancreas of the freshwater prawn *Macrobrachium australiense*. Results indicated that the gill expresses arginine kinase, NKA, VHA and CA more highly than the antennal gland and hepatopancreas (Moshtaghi et al., 2016). If we compare the gene and protein expression levels of gills and antennal glands between Ocypodoidea and other superfamilies species, we will realize how the antennal gland supports homeostasis and what the differences are in ion regulatory mechanisms between gills and the antennal gland among different species in various environments.

## Conclusion

We can broaden our understanding of diverse ion regulatory patterns among brachyuran when we consider the combined effects of phylogenetic relationships and environmental properties. The complex interactions among the antennal gland, gill and lung-like structure and can somehow be inferred using crab phylogenies and habitats. Statistical methods with phylogenetic correction, including PGLS and Moran’s *I*, can give us a more precise results in multi-species comparison. PGLS analysis in present review showed that the NKA activity in antennal gland is correlated to the urine-hemolymph ratio for Na^+^ in crabs under hypo-osmotic stress. Only a few studies address the structure and the distribution of ion regulatory proteins in the antennal gland among brachyurans, and there are still gaps in the linkage between structural and functional differences in crab antennal glands. It is possible to conduct the trait evolution analysis of ion regulation in a more integrative way by including a number of ion regulatory proteins in different organs among species—for example, using RNA sequencing or proteomics method to detect the expression difference of ion regulatory proteins and analyzing the patterns in the phylogenetic tree of crabs.
